# Performance Modeling and Cost Analysis of a Pilot-Scale Reverse Osmosis Process for the Final Purification of Olive Mill Wastewater

**DOI:** 10.3390/membranes3040285

**Published:** 2013-10-11

**Authors:** Javier Miguel Ochando-Pulido, Gassan Hodaifa, Maria Dolores Victor-Ortega, Antonio Martinez-Ferez

**Affiliations:** 1 Department of Chemical Engineering, University of Granada, 18071 Granada, Spain; E-Mails: marailo_21@hotmail.com (M.D.V.-O.); amferez@ugr.es (A.M.-F.); 2 Department of Molecular Biology and Biochemical Engineering, University of Pablo de Olavide, 41013 Seville, Spain; E-Mail: ghodaifa@upo.es

**Keywords:** olive mill wastewater, advanced oxidation processes, membrane processes, reverse osmosis, wastewater reclamation, modelization

## Abstract

A secondary treatment for olive mill wastewater coming from factories working with the two-phase olive oil production process (OMW-2) has been set-up on an industrial scale in an olive oil mill in the premises of Jaén (Spain). The secondary treatment comprises Fenton-like oxidation followed by flocculation-sedimentation and filtration through olive stones. In this work, performance modelization and preliminary cost analysis of a final reverse osmosis (RO) process was examined on pilot scale for ulterior purification of OMW-2 with the goal of closing the loop of the industrial production process. Reduction of concentration polarization on the RO membrane equal to 26.3% was provided upon increment of the turbulence over the membrane to values of Reynolds number equal to 2.6 × 10^4^. Medium operating pressure (25 bar) should be chosen to achieve significant steady state permeate flux (21.1 L h^−1^ m^−2^) and minimize membrane fouling, ensuring less than 14.7% flux drop and up to 90% feed recovery. Under these conditions, irreversible fouling below 0.08 L h^−^^2^ m^−^^2^ bar^−^^1^ helped increase the longevity of the membrane and reduce the costs of the treatment. For 10 m^3^ day^−1^ OMW-2 on average, 47.4 m^2^ required membrane area and 0.87 € m^−3^ total costs for the RO process were estimated.

## 1. Introduction

**Figure 1:**
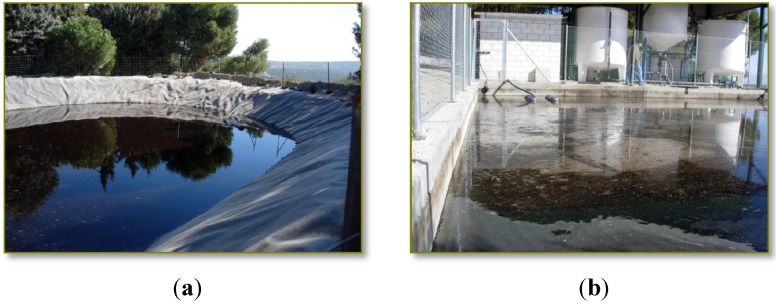
Olives washing wastewater (OWW) (**a**) and olive oil washing wastewater (OOW) (**b**) storage and evaporation lagoons.

Two principal wastewater streams are nowadays by-produced in olive mills working with the two-phase system during the production process of olive oil: olives washing wastewater (OWW), which is produced during the olive-fruit washing procedure in the washing machines, and olive oil washing wastewater (OOW), which is derived in the olive oil extraction process during the washing of the olive oil in the vertical centrifuges (Figure 1). The treatment of the liquid effluents generated during olive oil production in olive mills, together called olive mill wastewater (OMW-2), is currently still a hard task, due to multiple factors [1,2].

At the present, an average-sized olive oil factory working in continuous mode leads to a daily amount of up to 10–15 m^3^ of OOW, in sum to 1 m^3^ day^−^^1^ of OWW. In order to get an idea of the dimensions of this, in Spain alone, this generates a total volume of more than 9 million m^3^ of OMW-2 per year. Furthermore, the pollutants load of these effluents is extremely variable, not only owing to the extraction process, but also to edaphoclimatic and cultivation parameters, the type of the olives, their quality and maturity, and other factors [1,3]. These factors, in addition to small size and widespread geographical dispersion of olive oil factories, establish important difficulties for the management of these hazard effluents.

These wastewaters are highly phytotoxic and hazardous for water bodies, soil and aquifers due to the presence of aromatic compounds and a wide range of other organic pollutants, such as phenols, tannins and long-chain fatty acids, which are not suitable to be biologically managed. Due to the resistance of OMW-2 to microbial degradation [3], biological treatment of OMW-2 is not applied on an industrial scale as it is not efficient. Within this context, advanced operation processes (AOPs) are required for the depuration of these bio-refractory wastewaters [4,5,6,7,8,9,10,11,12,13,14,15,16].

Moreover, these effluents also exhibit high saline toxicity, and thus very significant electroconductivity (EC) values owing to the presence of a high concentration of inorganic compounds among which chloride, sulfate and phosphoric salts of potassium, calcium, iron, magnesium, sodium, copper and traces of other elements are common characteristics [1]. This fact also makes it necessary to resort to advanced separation technologies in order to attempt complete depuration of OMW-2, since it is not viable to abate the high concentration of dissolved monovalent and divalent ions by conventional physicochemical treatments. In this regard, as regulations become more stringent, membranes technology offers many advantages compared with other separation processes [17,18].

**Figure 2:**
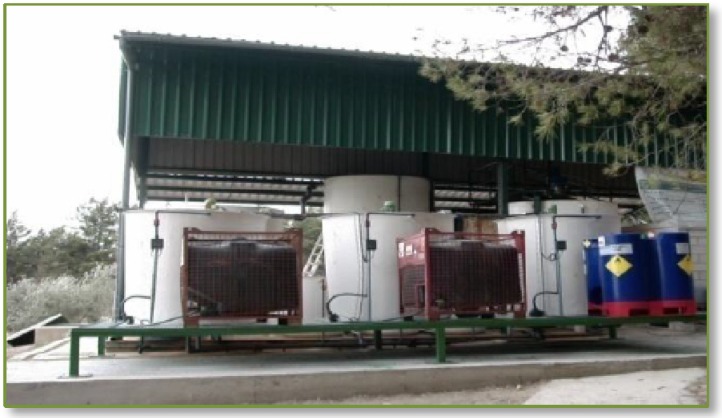
The secondary treatment plant set-up in an olive oil mill located in Jaén (Spain).

A secondary treatment for olive mill wastewater coming from factories working with the two-phase olive oil production process (OMW-2) has been recently set-up and transferred to an industrial scale in an olive oil mill in the premises of Baeza (Jaén, Spain) by the *Chemical and Biochemical Processes Technology* Research Group of the University of Granada (Granada, Spain) (Figure 2). The secondary treatment comprises Fenton-like oxidation, flocculation and filtration through olive stones. Fenton’s process appears to be the most economically advantageous AOP since it may be conducted at ambient temperature and pressure conditions, and also due its equipment simplicity and operational ease. Furthermore, the use of olive stones boosts the cost-effectiveness of the secondary treatment process as it is an abundant and renewable agricultural residue available at zero cost.

In this research work, ulterior purification of OMW-2 was intended with the goal of closing the loop of the industrial production process, pursuing the quality standards for reuse of the purified effluent in the proper olive washing machines. With this intention, modelization of the performance and preliminary cost analysis of a final reverse osmosis (RO) process was studied on a pilot scale.

## 2. Experimental Section

### 2.1. Analytical Methods

Analytical grade reagents and 99% purity chemicals were used for the analytical procedures, applied at least in triplicate. Chemical oxygen demand (COD), total suspended solids (Tss), total phenols (TPh), total iron, electroconductivity (EC) and pH measurements were carried out in the raw OMW-2 stream and in the treated effluent at the end of each depuration step following standard methods [19].

Chemical oxygen demand (COD) was determined by the photometric determination of the concentration of chromium (III) after 2 h of oxidation with potassium dichromate/sulfuric acid/silver sulfate at 148 ± 0.5 °C (German standard methods DIN 38 409-H41-1 and DIN ISO 15 705-H45) [19]. At the time of the determination of COD, manganese oxide was used to remove residual hydrogen peroxide which remained unreacted in samples taken from the reactor.

To determine the total suspended solids concentration (Tss), wastewater samples were filtered through 1.6 μm standard GF/F glass fiber filters. The residue retained on the filter was dried in an oven at 105 ± 0.5 °C until constant weight was observed. The increase in weight of the filter represents the Tss. Ashes correspond to the mineral salts remaining after the waste sample was calcined further at 600 ± 0.5 °C for 3 h [19].

Total phenols and phenol derivatives were analyzed by reaction with a derivative thiazol, giving a purple azo dye which was determined photometrically at 475 nm (Standard German methods ISO 8466-1 and DIN 38402 A51) [19].

EC and pH measurements were performed with a Crison GLP31 conductivity-meter and a Crison GLP21 pH-meter, with autocorrection of temperature. A Helios Gamma UV-visible spectrophotometer (Thermo Fisher Scientific) served for COD, TPh and total iron measurements. Effluent samples were diluted when necessary with MilliQ^®^ water for their analysis, whereas samples of RO permeate were analyzed directly without dilution.

Ionic concentrations were analyzed in the raw OMW-2, in the effluent exiting the secondary treatment (OMWST-2) as well as in the permeate stream of the final RO membrane stage with a Dionex DX-120 ion chromatograph as described in previous works [20,21].

Total iron concentration was measured by reducing all iron ions to iron ions (II) in a thioglycolate medium with a derivative of triazine, forming a reddish-purple complex photometrically determined at 565 nm (Standard German methods ISO 8466-1 and German DIN 38402 A51) [19].

### 2.2. The OMW-2 Stream

Samples of both OWW and OOW were collected during winter months from various olive oil factories working with the modern two-phase olive oil extraction process in the Andalusian province of Jaén (Spain). The physicochemical characterization of the raw OWW and OOW samples are reported in Table 1.

**Table 1:** Physicochemical characterization determined of raw OWW and OOW.

Parameters	Raw OWW	Raw OOW
pH	6.3	4.9
EC, mS cm^−1^	1.5	2.7
BOD_5_, mg O_2_ L^−1^	0.50	0.79
COD, mg O_2_ L^−1^	0.7	7.4
Total phenols, mg L^−1^	3.7	561.5
Moisture, %	99.7	99.3
Total solids, %	0.27	0.6
Organic substances, %	0.10	0.49
Ashes, %	0.17	0.11

**Figure 3:**
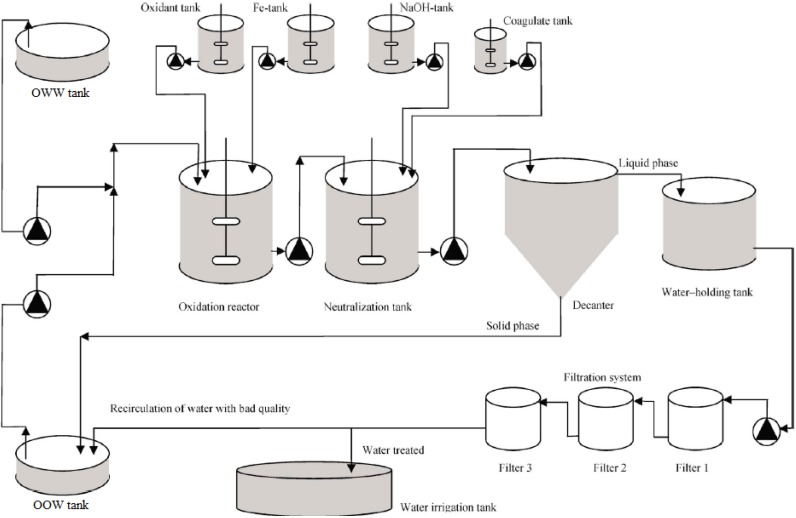
Scheme of the industrial treatment plant for OMW-2 located in Jaén (Spain).

Firstly, OWW and OOW samples were rapidly analyzed in the laboratory. Afterwards, both effluents were mixed in 1:1 (*v*/*v*) proportion in order to stabilize the mean value of the organic concentration and avoid sensible fluctuations in the COD parameter of the stream entering the treatment system (OMW-2) (Table 2). OMW-2 stream was conducted to the secondary treatment already set-up on an industrial scale by the *Chemical and Biochemical Processes Technology* Research Group of the University of Granada (Granada, Spain). The secondary treatment consists basically in an AOP based on Fenton’s reagent. In the industrial plant, the use of the cheaper ferric (FeCl_3_) catalyst (Fenton-like oxidation) instead of ferrous ones highly improves the cost-effectiveness of the process [5,6]. Following the oxidation reactor, flocculation-sedimentation and filtration in series ensure recovery of the catalyst [22,23]: the iron-rich sludge sedimented in the decanter is recirculated to the OWW tank (Figure 3) to reduce the catalyst consumption [22], whereas olive stones permit eliminating the residual iron which is known to be especially harmful for polymeric membranes [24]. In this regard, olive stones represent an abundant and renewable agricultural residue available at zero cost. A scheme of the industrial-scale secondary treatment is given in Figure 3, and the physicochemical composition of the raw OMW-2 stream and the effluent exiting the secondary treatment (OMWST-2) is reported in Table 2.

**Table 2:** Olive mill wastewater (OMW-2) characterization at the outlet of each secondary treatment stage.

Parameters	Raw OMW-2	OMW-2 at the outlet of the secondary treatment
pH	5.7	7.7
EC, mS cm^−1^	2.3	3.43
Tss, mg L^−1^	60.1	13.1
COD, mg O_2_ L^−1^	4050.5	150.8
Total phenols, mg L^−1^	282.6	0.4
Total iron, mg L^−1^	7.9	0.03
Cl^−^, mg L^−1^	554.5	990.9
Na^+^, mg L^−1^	486.2	718.6

### 2.3. Membranes Pilot Plant

**Figure 4:**
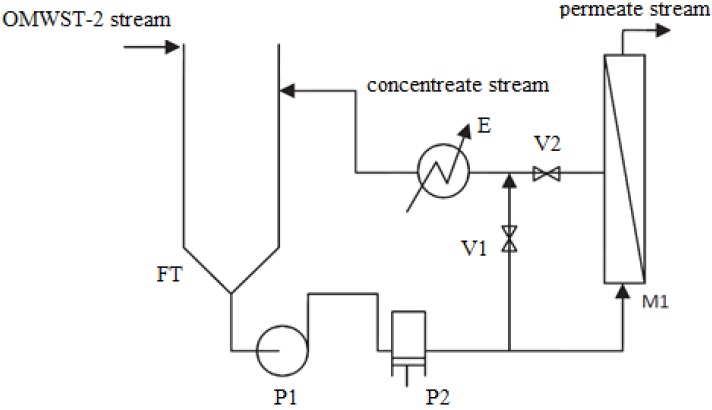
Membrane filtration pilot plant flow scheme. FT: feed tank; P_1_: booster pump; P_2_: volumetric pump; V_1_: bypass regulation valve; V_2_: concentrate regulation valve; E: plate heat exchanger; M_1_: membrane housing provided with SW membrane.

The research study on the final RO membrane operation was conducted in a pilot plant provided with a 100 L feed tank (FT), where the pretreated feedstock was loaded through a feed line, and a pumping system consisting of a centrifugal booster pump (P_1_) and a volumetric piston pump (P_2_) that served to drive the OMWST-2 stream to the spiral-wound (SW) membrane module fitted in housing M_1_ (Figure 4).

Tangential-flow RO experiments were performed in semicontinuous operation mode (diafiltration): the permeate stream was collected whereas the concentrate stream was recycled back the feed tank while entering new fresh OMWST-2 feedstock to the membrane system.

The selected RO membrane was a commercial spiral-wounded thin-film composite (TFC) membrane provided by GE Water and Process Technologies (Minnetonka, MN, USA), consisting of a polyamide active layer on a polysulfone ultrafiltration support, with an active area of 2.5 m^2^. Nominal characteristics of the selected virgin RO membrane and of the spacer are reported in Table 3.

**Table 3:** Membrane specifications.

Parameters	Parametric value
Membrane model	SC series (RO)
Nominal salt rejection, %	98.9
Effective surface area, m^2^	2.5
Permeability, L h^−1^ m^−2^ bar^−1^	1.4 ± 0.2
Configuration	Spiral-wound
Chemical composition	TFC aromatic polyamide/polysulfone
Surface nature	Hydrophilic
Maximum pressure, bar	40
Maximum temperature, °C	90
pH range	1–11
Spacer configuration	45 mil parallel
Spacer material	Polymeric
Spacer mesh diameter, mm	2

The desired operating pressure over the membrane and the feed flow rate to the module could be adjusted by acting on the regulation valves V_1_ and V_2_, which are shown in Figure 4, with an accuracy of 0.5 bar and 10 L h^−1^ respectively. The V_1_ valve was used for by-pass whereas the V_2_ valve served for regulating the flow rate of the concentrate stream, which was recirculated to the feedwater tank. Operating pressure and feed flow rate were respectively measured by analogue manometers and a turbine flow meter, whereas permeate flux was calculated during operation time by weighing the mass of collected permeate on a precision electronic mass balance (AX-120 Cobos, 0.1 mg accuracy). In all experiments, operating temperature was controlled at a fixed value equal to ambient temperature (22 °C) by means of a plate heat exchanger (E) (see Figure 4), regulated automatically (T_set point_ ± 0.1 °C) via a proportional–integral–derivative (PID) electronic temperature controller (Yokogawa model UT100) and a magnetic valve in the temperature-controlling loop.

## 3. Results and Discussion

The objective of this work was to close the loop of the industrial production process of an olive mill working with the two-phase extraction procedure in Baeza (Jaén, Spain). With this intention, optimization of a final RO operation to achieve the quality standards for reuse of the purified OMW-2 effluent in the proper olive washing machines was examined. Performance of the RO membrane module was modelized, including permeate flux and membrane rejection as function of the main operating variables, *i.e.*, operating pressure and tangential velocity. Finally, membrane fouling and life-time service as well as simplified economical analysis of the RO process was studied.

Firstly, the effect of the net driving pressure of the system on the permeate flux of the selected RO membrane was studied by fitting the experimental data to the solution-diffusion model:

*J_p_* = *K* ∙ (Δ*P* − Δ*π*) (1)

where *J_p_* is the permeate flux (L h^−1^ m^−2^), *K* represents the permeability of the membrane (L h^−1^ m^−2^ bar^−1^) and Δ*P −* Δ*π* (bar) is the net pressure (*P*_TM_), that is the difference between the applied pressure (Δ*P*) and the osmotic pressure (Δ*π*) of the influent entering the RO membrane module.

The data regarding the dependence of the membrane permeability on the net driving pressure and tangential velocity are reported in Table 4 in the form of pure water permeability (*K*_w_) and permeability with the OMWST-2 effluent (*K*), obtained for ambient operating temperature (22 °C). The osmotic pressure of OMWST-2, equal to 1.5 bar at 22 °C, was calculated by means of Van’t Hoff equation *π_f_* = Σ*c_i_*_,*f*_·RT, where *c_i_*_,*f*_ is the feed molar concentration of ion *i* (mol L^−1^), R is the ideal gas constant (8.314 J K^−1^ mol^−1^) and T is the absolute temperature (K). 

**Table 4:** Permeability coefficients and resistances of the selected reverse osmosis (RO) membrane.

*v_t_*, m s^−1^	*N*_Re_	*K*_w_, L h^−1^ m^−2^ bar^−1^	R_m_, m^−1^	*K*, L h^−1^ m^−2^ bar^−1^	R_CP_, m^−1^
2.55	1.3 × 10^4^	1.41 ± 0.1	2.6 × 10^14^	0.86 ± 0.1	1.9 × 10^14^
5.09	2.6 × 10^4^	1.41 ± 0.1	2.6 × 10^14^	0.90 ± 0.1	1.4 × 10^14^

Notes: Operating conditions: 3–35 bar, 22 °C; *v_t_*: tangential velocity; *N*_Re_: Reynolds number.

Results withdrawn show a value of the pure water permeability coefficient (*K*_w_) of 1.41 L h^−1^ m^−2^ bar^−1^, whereas *K* measured with the secondary-treated effluent (OMWST-2) was equal to 0.86 L h^−1^ m^−2^ bar^−1^ upon turbulent regime over the membrane (tangential velocity equal to *v_t_* = 2.55 m s^−1^, corresponding to Reynolds number *N*_Re_ = 1.3 × 10^4^). The striking difference in the permeability coefficients between pure water and OMWST-2 (36.2%– 39.0% for *N*_Re_ = 1.3 × 10^4^ − 2.6 × 10^4^) shall be attributed to concentration polarization and fouling resistance taking place on the boundary region of the RO membrane (Table 4) [25,26]. A guided focus on Table 4 permits us to note that reduction of the concentration polarization (*R*_CP_) on the RO membrane equal to 26.3% was provided upon an increment of the turbulence on the membrane to values of *N*_Re_ = 2.6 × 10^4^.

Secondly, membrane fouling was examined by calculating the fouling index (*b*) at every operating condition during diafiltration run time. With this purpose, the experimental permeate flux data were fitted with the threshold flux equation proposed by Field and Pearce (2011) [27]:

*J_t_* = *J*_th_ + (*J*_0_ − *J*_th_) ∙ *e^−bt^* − *a* ∙ *t* (2)

where the variable *b* is the reversible fouling index (h^−l^) and *a* the long-term irreversible fouling parameter (L h^−^^1^ m^−^^2^ bar^−^^1^), *J*_0_ is the initial permeate flux (L h^−^^1^ m^−^^2^) that can be easily calculated through Equation (1), *J_t_* is the permeate flux at a given time (L h^−^^1^ m^−^^2^) and *J*_th_ represents the threshold flux (L h^−^^1^ m^−^^2^), being *b*, *a* and *J*_th_ fitting parameters obtained simultaneously by a non-linear parameter estimation method [28,29,30,31]. The *b* index takes into account the fouling developed on the membrane during operation time that can be periodically washed to recover the initial permeability (*K*) of the membrane [32,33], meanwhile the *a* parameter accounts for the irreversible fouling unavoidably attained on the membrane that makes the initial permeability to steadily decrease during the membrane service lifetime in a minor or major degree, thus establishing the module substitution periodicity.

Results are given in Table 5, showing that medium operating pressure should be chosen (25 bar) upon turbulent regime over the membrane (5.09 m s^−1^, that is *N*_Re_ = 2.6 × 10^4^) to achieve significant steady state permeate flux (21.1 L h^−^^1^ m^−^^2^) and minimize membrane fouling (*b* index equal to 0.32), ensuring less than 14.7% permeate flux drop and up to 90% feed recovery. 

**Table 5:** Results determined for the RO performance at several operating conditions.

*v_t_*, m s^−1^	*P*_TM_, bar	*T*, °C	*J*_0,_ L h^−1^ m^−2^	*J*_ss,_ L h^−1^ m^−2^	−Δ*J_p_*_,_ %	*b*, h^−1^	*Y*, %
2.55	35	22	32.1	n.o.	24.7	1.57	40
2.55	25	22	21.8	15.2	20	0.67	85
2.55	15	22	15.7	12.4	20.5	1.03	50
5.09	25	22	24.6	21.1	14.7	0.32	90

Notes: *v*_t_: tangential velocity; *J*_ss_: steady-state permeate flux; −Δ*J*_p_: permeate flux loss at the end of experiment; *R*_EC_, *R*_COD_: conductivity and COD rejection; *b*: fouling index; *Y*: volume recovery; n.o.: not observed.

Finally, selectivity of the selected RO membrane was modelized by means of the Spiegler-Kedem model:

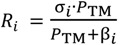
(3)
which allows prediction of the rejection efficiency of the solute *i* (*R_i_*) as function of the net pressure (*P*_TM_ = Δ*P* − Δ*π*) as well as two parameters: the first is the reflection coefficient σ*_i_* that indicates the maximum rejection capability of the RO membrane (0 < σ*_i_* < 1) and the second is β*_i_* which is a fitting constant [32,33]. Both parameters, estimated simultaneously by non-linear parameter estimation method, are reported in Table 6 together with the rejection efficiency values achieved for EC and COD [28,29]. Accurate prediction of the experimental EC and COD rejection was attained by the Spiegler-Kedem model, and regression coefficients *R*^2^ equal to 0.99 and 0.98 were obtained respectively. Minimum rejection coefficients for COD and EC equal to 99.1% and 98.1% were measured in the permeate stream. This ensured that the parametric values were maintained below standard limits established to reuse the regenerated effluent in the olives washing machines of the olive oil production process.

**Table 6:** Coefficients for electroconductivity (EC) and chemical oxygen demand (COD) rejection.

Parameter	Rejection, %	σ*_i_*	β*_i_*, bar
EC	99.1–99.8	1	0.48
COD	98.1–99.2	1	0.63

A simplified economical balance of the treatment process was performed based on a daily amount of 10 m^3^ OMW-2 on average. In our former studies [6,22], the secondary treatment process was presented as an economically advantageous AOP, providing high mineralization of the recalcitrant organic matter present in the raw effluent [34]. Estimated operating costs of approximately 2.4 € m^−3^ for conductive-diamond electrochemical oxidation, 8.5 € m^−3^ for ozonation and 0.7 € m^−3^ for Fenton-like process highlight the latter as the most cost-effective one. Furthermore, filtration through olive stones is costless [23].

Under the best operating conditions studied for the final RO operation, significant steady-state permeate flux production was attained (21.1 L h^−1^ m^−2^) and the constant fouling parameter *a* was estimated to be −0.08 L h^−2^ m^−2^ bar^−1^. The *a* parameter takes into account the irreversible fouling irretrievably attained on the RO membrane that steadily reduces its virgin permeability and therefore has direct implications in the module substitution periodicity. A membrane lifetime over 5 years can be ensured for a mean daily operating time equal to 10 h and taking into consideration that the olive oil campaign, when the OMW-2 is generated, lasts 90 days a year on average. The minimum required membrane area is equal to 47.4 m^2^ for the daily volume of OMW-2 treated. This implies the need of two RO membrane modules of 32 m^2^ surface each. The modules would work in parallel configuration: one in service, whereas the other would be in cleaning protocol when the diafiltration cycle finishes. This means an overdesign of 26% of the required membrane surface. Energy costs were fixed at 0.10 € kWh^−1^, whereas membrane module and housing were equal to 22 € m^−2^ and 380 €. Pumps, piping and instrumentation of the RO membrane plant represent approximately 50% of the membrane housing costs, whereas facility space needs and services represent 15% of total costs [35,36].

**Figure 5:**
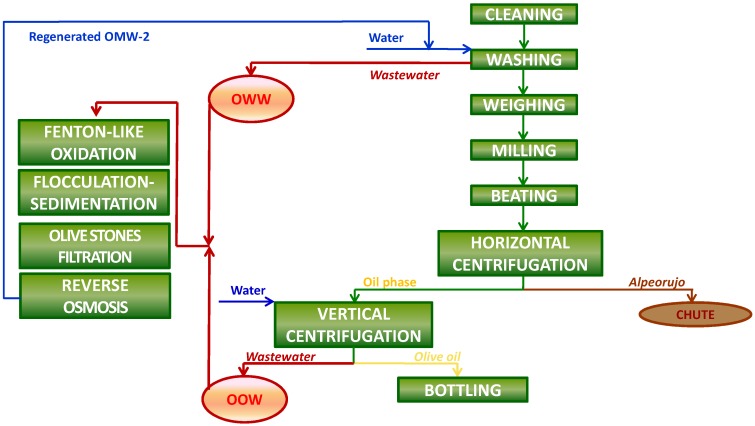
Integrated olive oil production process.

**Figure 6:**
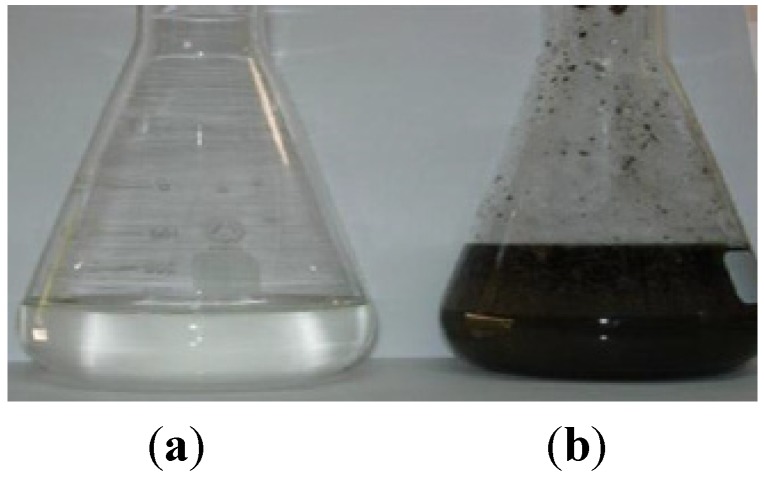
Final treated OMW-2 (**a**) in contrast with raw OMW-2 (**b**).

Finally, the total expenses of the whole integrated OMW-2 treatment process, taking into account the final purification of the effluent by the selected TFC RO membrane and the secondary treatment, are given in Table 7, and the physicochemical characteristics of the final permeate stream are reported in Table 8. The proposed integrated treatment permits us to close the loop of the olive oil production process (Figure 5), rending it environmentally respectful (Figure 6) and boosting its cost-effectiveness.

**Table 7:** Cost evaluation of RO operation and whole integrated process.

Parameter	Cost, € m^−3^
Membrane	0.25
Housing and piping	0.22
Current	0.40
*Total costs of RO operation*	*0.87*
*Total costs of the integrated process*	*1.57*

**Table 8:** Physicochemical composition of permeate stream.

Parameter	Value in permeate stream
pH	7.6–7.7
EC, µS cm^−1^	95.0–97.0
Tss, mg L^−1^	0
COD, mg L^−1^	2.3–3.7
Total phenols, mg L^−1^	0
[Fe]_Total,_ µg L^−1^	0
[Cl^−^], mg L^−1^	15.5–20.7
[Na^+^], mg L^−1^	11.1–16.7

## 4. Conclusions

Performance modelization and preliminary cost analysis of a final reverse osmosis (RO) treatment was examined on a pilot scale for ulterior purification of the effluents generated by olive oil factories working with the two-phase olive oil extraction procedure. The goal was closing the loop of the industrial production process of an olive oil mill in the premises of Jaén (Spain), where a secondary treatment comprising Fenton-like oxidation, flocculation and filtration through olive stones has been successfully set-up.

Reduction of concentration polarization on the RO membrane equal to 26.3% was provided upon increment of the turbulence over the membrane to values of Reynolds number equal to 2.6 × 10^4^. Medium operating pressure (25 bar) helped achieve significant steady state permeate flux (21.1 L h^−1^ m^−2^) and minimize membrane fouling, ensuring less than 14.7% flux drop and up to 90% feed recovery. Under these conditions, less than 0.08 L h^−^^2^ m^−^^2^ bar^−^^1^ irreversible fouling was measured, increasing the longevity of the membranes and reducing the capital and operating costs of the treatment. Finally, 47.4 m^2^ required membrane area and 0.87 € m^−3^ total costs were estimated for the RO process on a daily basis of 10 m^3^ OMW-2. The proposed integrated process permits us to close the loop of olive oil production, rending it environmentally respectful and boosting its cost-effectiveness.
